# Influence of the geometric and material properties of lumbar endplate on lumbar interbody fusion failure: a systematic review

**DOI:** 10.1186/s13018-022-03091-8

**Published:** 2022-04-10

**Authors:** Yihang Yu, Dale L. Robinson, David C. Ackland, Yi Yang, Peter Vee Sin Lee

**Affiliations:** 1 Department of Biomedical Engineering, University of Melbourne, Parkville, VIC 3010 Australia; 2 Department of Orthopaedics, Royal Melbourne Hospital, Parkville, VIC 3052 Australia

**Keywords:** Lumbar interbody fusion, Lumbar endplate, Interbody cage, Cage subsidence, Cage migration, Non-union

## Abstract

**Background:**

Lumbar interbody fusion (LIF) is an established surgical intervention for patients with leg and back pain secondary to disc herniation or degeneration. Interbody fusion involves removal of the herniated or degenerated disc and insertion of interbody devices with bone grafts into the remaining cavity. Extensive research has been conducted on operative complications such as a failure of fusion or non-union of the vertebral bodies. Multiple factors including surgical, implant, and patient factors influencing the rate of complications have been identified. Patient factors include age, sex, osteoporosis, and patient anatomy. Complications can also be influenced by the interbody cage design. The geometry of the bony endplates as well as their corresponding material properties guides the design of interbody cages, which vary considerably across patients with spinal disorders. However, studies on the effects of such variations on the rate of complications are limited. Therefore, this study aimed to perform a systematic review of lumbar endplate geometry and material property factors in LIF failure.

**Methods:**

Search keywords included ‘factor/cause for spinal fusion failure/cage subsidence/cage migration/non-union’, ‘lumbar’, and ‘interbody’ in electronic databases PubMed and Scopus with no limits on year of publication.

**Results:**

In total, 1341 articles were reviewed, and 29 articles were deemed suitable for inclusion. Adverse events after LIF, such as cage subsidence, cage migration, and non-union, resulted in fusion failure; hence, risk factors for adverse events after LIF, notably those associated with lumbar endplate geometry and material properties, were also associated with fusion failure. Those risk factors were associated with shape, concavity, bone mineral density and stiffness of endplate, segmental disc angle, and intervertebral disc height.

**Conclusions:**

This review demonstrated that decreased contact areas between the cage and endplate, thin and weak bony endplate as well as spinal diseases such as spondylolisthesis and osteoporosis are important causes of adverse events after LIF. These findings will facilitate the selection and design of LIF cages, including customised implants based on patient endplate properties.

**Supplementary Information:**

The online version contains supplementary material available at 10.1186/s13018-022-03091-8.

## Background

Lumbar degenerative disc disease (DDD) is an intervertebral disc pathology characterised by the deterioration or breakdown of one or more discs between the lumbar vertebrae. It is strongly associated with lower back pain in the younger population (younger than 50 years) [1].

In disc degeneration cases associated with leg and radicular pain, the ‘gold standard’ for surgical treatment of severe lower back pain caused by DDD is lumbar interbody fusion (LIF), wherein the disc is replaced by one or more interbody cages and bone grafts to support the intervertebral space and enable fusion between adjacent vertebrae [2].

Despite the widespread acceptance of LIF, fusion is not achieved in all patients. A systematic review has shown that non-fusion rates for LIF at L5/S1 level ranged from 0.2% to 21.0% in 22 years (from 1992 to 2014) [3]. Although symptomatic patients with failed fusion can undergo revision surgery [4, 5], the complication rates of revision surgeries are significantly higher [6, 7].

The interbody cage acts as a spacer between the affected vertebrae, and it plays a crucial role in LIF to restore disc height and promote bony ingrowth [8]. Titanium alloy and polyetheretherketone (PEEK) are the most common materials used in interbody cages, where titanium alloy can stimulate bony ingrowth and PEEK material can mimic the density and stiffness of vertebrae [9, 10]. Interbody cages are available in varying sizes and shapes to fit the intervertebral space, including cylindrical, rectangular, wedge-shaped, or banana-shaped [11, 12]. The cage position on the endplate is another LIF variable, and bilateral, unilateral, and anterior cage positioning are commonly used [13]. Multiple factors are considered in designing an intervertebral cage, such as geometry, material properties, ease and safety of intraoperative insertion. The material, shape, and size of interbody cages have been specifically developed to allow for an optimum fit between the vertebrae within the previous disc space and to promote bone growth across the disc space leading to fusion. The cages aim to conform to the geometry and material properties of the bony endplate for its insertion and fitting into different positions of the intervertebral space. Therefore, understanding variations in geometric and material properties across the endplates of the lumbar spine is essential for optimum spinal interbody cage design.

In the asymptomatic spine, the surfaces of lumbar endplates are generally concave, with the cranial endplate relative to the disc exhibiting greater concavity compared with the caudal endplate [14]. The endplate concavity increases from L1 to L5, and the greatest area of concavity of each endplate is located in the middle or posterior region of the vertebral body [15]. For each intervertebral disc, compared to the cranial endplate, the caudal endplate has a larger surface area, and the surface area is larger in males and mostly proportional to the intervertebral space [14, 16, 17]. The disc angle (lordotic angle) between two adjacent endplate surfaces for the standing posture increases toward the lower level of vertebrae [15]. This can affect the inclination of the interbody cages used at the lumbar level.

The material properties of the lumbar endplate, specifically its bone mineral density (BMD), vary along the spine, wherein cranial endplates of intervertebral spaces usually have a higher BMD and are stronger and stiffer compared with the caudal endplates [18, 19]. These spatial differences in endplate properties have been attributed to the greater rates of vertebral fractures at the caudal endplates [20–22]. Furthermore, the peripheral regions of lumbar endplate surfaces are of greater strength compared with the central areas [19, 23], which is in accordance with the respective thickness between these areas [24]. Considerable variations in endplate geometry and material properties also exist in terms of individuals, sex, ageing, and degeneration [15, 16]. For example, endplate thickness and surface area correlate with age [15, 16]. Furthermore, severe intervertebral disc degeneration significantly decreases the endplate concavity and endplate area [16].

Previous research regarding LIF failure suggests that outcomes of fusion surgery are related to spinal segment properties, device properties, and surgeon experience [25]. However, to date, to the best of our knowledge, no study has reviewed the endplate-related risk factors for fusion failure. To bridge this gap in knowledge, this systematic review aimed to identify and compare the lumbar endplate geometric and material factors that contribute to LIF failure to help address implant-related complications in the development of customised spinal implants.

## Methods

### Search strategy

A systematic review was performed in the PubMed and Scopus databases to identify studies that describe influence of lumbar endplate-related factors on spinal fusion failures or complications. A search strategy was used to minimise possible missing of relevant studies:

(factor for) OR (cause for) AND (spinal fusion failure) OR (cage migration) OR (cage subsidence) OR (non-union) AND (lumbar) AND (interbody).

Only English language articles were searched, and there were no restrictions on the year of publication.

### Selection strategy

**Fig. 1:**
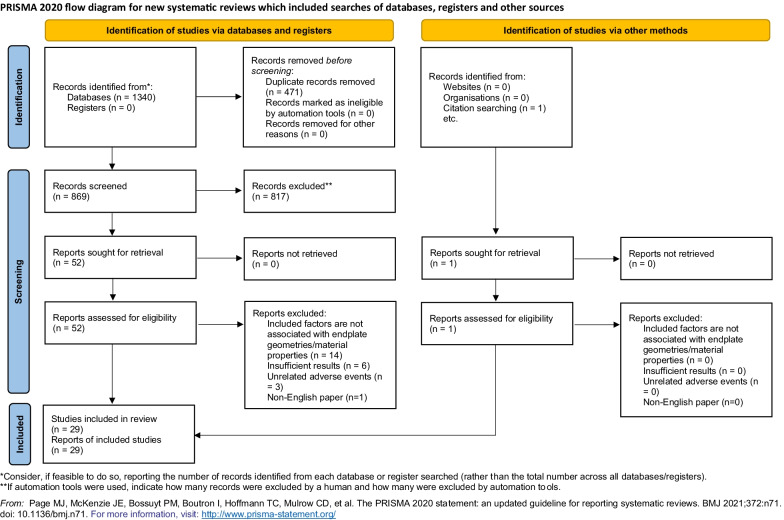
PRISMA flow diagram for systematic review

The search and selection process ended on 14/09/2021, and the whole process was conducted in accordance with the Preferred Reporting Items for Systematic Reviews and Meta-Analyses (PRISMA) statement [26]. The Sample, Phenomenon of Interest, Design, Evaluation, and Research Type (SPIDER) tool was used to structure the research question, where sample (S) is the patients who underwent LIF, phenomenon of interest (PI) is fusion failure, design (D) is observational studies or survey, evaluation (E) is the influence of the geometric and material properties of lumbar endplate, and research type (R) is qualitative or quantitative or mixed [27]. Selection process was done in the Covidence Systematic Review Software [28] to retain records for each step. Selection criteria were applied to the resultant articles after removing duplicates (Table 1). The inclusion criteria were based on surgery type, implant involvement, surgery outcome category, and relevance of factors. The exclusion criteria were also applied to the resultant articles and were based on the study type (e.g. animal studies and computational studies were excluded). Detailed reasons for setting up these exclusion criteria are provided in Additional file 1. The references of the selected articles were subsequently screened to include more articles in this systematic review. The PRISMA flow diagram is provided in Fig. 1, and the details of PRISMA checklist are provided in Additional file 2.

**Table 1:** Details of selection criteria

Inclusion criteria	Exclusion criteria
Lumbar interbody fusion	Animal studies
Interbody cage involved	Finite element (computational) studies
Adverse events after fusion	Rare case studies
Risk factors for fusion failure/adverse events	Previous systematic review/meta-analysis
Factors associated with endplate geometries/material properties

### Quality assessment

Since most of the selected articles were observational studies and the results belonged to both randomised or non-randomised studies, two well-established quality assessment checklists appropriate for these study types were considered: the Strengthening the Reporting of Observational Studies in Epidemiology (STROBE) statement checklist [29] and the Downs and Black checklist [30]. Based on their principles, a customised checklist for assessing the quality of the included articles was created. There were 12 requirements in this checklist focusing on the background, aims, methodology, results, discussion, and limitations of the evaluated articles. Each requirement was graded as clearly reported, partially reported, or not reported, and was associated with a score of 2, 1, or 0, respectively, which was subsequently added to the total. The quality assessment was performed by two reviewers independently, and any disagreement of opinion was resolved by discussion. Articles with scores $\ge$ 20 were defined as high-quality studies, those with scores ranging from 14 to 20 were defined as moderate-quality studies, and those with scores less than 14 were defined as low-quality studies. The customised checklist is provided in Additional file 1: Table S2.

### Data analysis

The included studies were divided into several groups based on the adverse events. Risk factors for adverse events in these studies were grouped based on demographics, diagnosis, anatomical and surgical factors, and cage specifications. Although the review aimed to identify risk factors related to endplate geometric and material properties, risk factors other than those included in the resultant studies were also analysed.

## Results

### Included articles

After removing duplicates, 869 articles were identified in the initial search. There were 52 articles deemed eligible after screening the title and abstract, of which 28 were included after full-text assessment based on the selection criteria [20, 25, 31–56]. The key reasons for excluding 24 articles [57–80] during the full-text screening were irrelevant risk factors, insufficient results, unrelated adverse events, and non-English paper. Reviewing citations in the 28 studies provided an additional article [81]. Twenty-nine studies were finally included in the current systematic review. The majority of the included articles were of moderate quality (n = 22), whereas five of high quality, and two of low quality (Additional file 1: Figure S1). A summary of the information of the included studies, such as the authors with year of publication, sample size, surgery type, adverse event categories, risk factors associated with endplate geometry or material property, and quality scores for the included articles is provided in Table 2. Details of quality assessment and available data of included articles were attached as additional files (Additional files 3, 4).

**Table 2:** Details of included articles

Authors	Sample size (patient number)	Surgery type	Adverse event category	Risk factors (associated with endplate)	Quality score
Abbushi et al. [31]	40 (80 cages)	PLIF	Cage subsidence	Cage position, cage shape	14
Amorim-Barbosa et al. [32]	165 (208 cages)	PLIF/TLIF	Cage subsidence	BMD, cage position	19
Aoki et al. [33]	125 (144 levels)	TLIF	Cage migration	Cage shape, size, disc height	17
Beutler and Peppelman [20]	104 (140 levels)	ALIF	Cage subsidence	Fusion level, cage position	15
Cho et al. [34]	86	PLIF	Cage subsidence, screw loosening	Osteoporosis	19
Duncan and Bailey [81]	102	TLIF	Cage migration	Screw fixation	17
Hu et al. [25]	953 (1559 cages)	TLIF	Cage migration	Cage position, disc height, fusion level, multi-level fusion	19
Jin et al. [35]	75	TLIF	Cage migration	Advanced age	16
Jones et al. [36]	347 (567 levels)	LLIF	Cage subsidence	BMD	21
Kim et al. [37]	104 (122 cages)	MITLIF	Cage subsidence	Fusion level, cage position	15
Kimura et al. [38]	1070 (1433 cages)	PLIF	Cage retropulsion	Fusion level, multi-level fusion, disc height, endplate shape	14
Konomi et al. [39]	78 (88 levels)	PLIF	Non-union	Advanced age	14
Le et al. [40]	140 (238 levels)	XLIF	Cage subsidence	Cage size	16
Lee et al. [41]	744 (1229 levels)	PLIF/TLIF	Cage retropulsion	Endplate shape, loosening of fixation	16
Li et al. [42]	286	PLIF/TLIF	Cage migration	Cage size, spondylolisthesis	14
Liu et al. [43]	215	TLIF	Cage migration	Cage shape, screw fixation	18
Marchi et al. [44]	74 (98 levels)	LLIF	Cage subsidence	Cage size	18
Mi et al. [45]	242	TLIF	Cage subsidence	Hounsfield units (BMD)	16
Okuyama et al. [46]	52	PLIF	Non-union	BMD	14
Pan et al. [47]	8	PLIF/TLIF	Cage retropulsion	Cage shape, fusion level	13
Park et al. [48]	784 (881 levels)	TLIF	Cage migration, subsidence, retropulsion	Osteoporosis, endplate shape, cage position	20
Singhatanadgige et al. [49]	114 (135 levels)	MITLIF	Cage subsidence	Cage position	18
Tempel et al. [50]	80	LLIF	Cage subsidence	BMD	14
Tohmeh et al. [51]	140 (223 levels)	XLIF	Cage subsidence	Cage size	21
Xi et al. [52]	68	LLIF	Cage subsidence	Hounsfield units (BMD)	21
Yao et al. [53]	96 (126 levels)	MITLIF	Cage subsidence	BMD, disc height	20
Zhao et al. [54]	512	TLIF	Cage migration	Cage size, shape, multi-level fusion	11
Zhou et al. [55]	145	TLIF	Cage subsidence	Irregular endplate shape	19
Zhou et al. [56]	121 (176 levels)	TLIF	Cage retropulsion	Irregular endplate shape, cage position	19

Surgery types in this table including anterior lumbar interbody fusion (ALIF), posterior lumbar interbody fusion (PLIF), transforaminal lumbar interbody fusion (TLIF), minimally invasive transforaminal lumbar interbody fusion (MITLIF), lateral lumbar interbody fusion (LLIF), and extreme lateral lumbar interbody fusion (XLIF)

### LIF failure

LIF failure involves loss of device fixation and non-union at the fusion site. Loss of fixation is associated with other adverse events such as implant subsidence and changes in implant position [82].

Adverse events after LIF were grouped into four general types—cage subsidence, cage migration, combined cage migration with subsidence, and non-union at the fusion level, and the incidence of these events reported by the included studies was recorded. The sample size and incidence rates for these adverse events are listed in Table 3.

**Table 3:** Summary of general adverse events after LIF along with number of studies, sample size, and complication rates

Adverse event	Number of studies	Sample size (patients)	Incidence rate (%)
Cage subsidence	15	2558	27.6
Cage migration	12	4995	3.1
Combined cage subsidence and migration	1	784	4.6
Non-union	2	130	10.0

The incidence rate for cage subsidence was calculated from cage number because of the incomplete data for patient number involved with cage subsidence

#### Cage subsidence

There were 15 studies that discussed cage subsidence after LIF [20, 31, 32, 34, 36, 37, 40, 44, 45, 49–53, 55], with qualities of moderate (*n* = 11) and high (*n* = 4). For the 2,558 cages pooled across these studies, 705 cages had subsidence (27.6%). Thresholds for identification of subsidence varied between 2 and 4 mm across studies [20, 31, 34, 37, 40, 49, 51, 53, 55]. Three studies classified subsidence into four grades based on the loss of disc height after LIF (Grade 0: a loss of 0–24% in disc height; Grade I: 25–49%; Grade II: 50–74%; Grade III: 75–100%), with a loss in disc height of 24% indicative of considerable cage subsidence [36, 44, 52].

#### Cage migration

There were 12 articles that analysed posterior cage migration after LIF (qualities: low/moderate/high, 2/9/1) [25, 33, 35, 38, 41–43, 47, 48, 54, 56, 81]. Of the 4995 patients included in these studies, 156 were identified with posterior migration (3.1%). Two alternative methods were used to identify posterior cage migration: when the posterior cage migration exceeded a 2-mm or 3-mm threshold [35, 43, 48] or when the cage moved beyond the posterior wall of adjacent vertebrae, which is also termed cage retropulsion [25, 33, 38, 41, 42, 47, 56, 81].

#### Non-union at the fusion level

The third adverse event after LIF discussed in this review was non-union between the vertebra adjacent to the cage. Only two studies, both of moderate quality [39, 46], assessed non-union after LIF, wherein the average non-union rate was 10.0% for 130 patients postoperatively after more than 2 years [39, 46]. These studies considered at least one of the following criteria to identify non-union: (1) any relative movement at the fusion site in lateral (flexion and extension) radiographs, (2) any visible gap between the endplate and spinal cage, and (3) absence of trabecular bone bridging. However, to the best of our knowledge, there is no consensus on the definition of non-union in the literature. For movement on lateral radiographs, Konomi et al. [39] only considered cases with lateral movements larger than 3°, whereas Okuyama et al. [46] included cases with any lateral movement.

#### Combined cage subsidence and migration

The only study to evaluate multiple adverse events following LIF was reported by Park et al. [48], who considered cases of migration with subsidence. This high-quality study evaluated 784 patients and found that this combined adverse event occurred in 36 patients (4.6%).

### Demographics and diagnosis

All included studies provided the age distribution, sixteen of them recorded sex distribution [25, 32, 34, 38, 41, 42, 44, 47–50, 52, 53, 55, 56, 81], seven included body mass index (BMI) [32, 41, 44, 49, 53, 55, 56], and three accounted for patients with osteoporosis [34, 41, 48]. The details are listed in Table 4.

**Table 4:** Patient demographics for included studies

Demographics	Patient number	Mean value/distribution
Age	7070	60.0 years (18.0–86.0 years)
Sex	4896	58.6%/41.4% (female/male)
BMI	1459	24.9 kg/m^2^ (20.8–32.0 kg/m^2^)
Osteoporosis	1614	11.0%/89.0% (osteoporosis/non-osteoporosis)

**Fig. 2:**
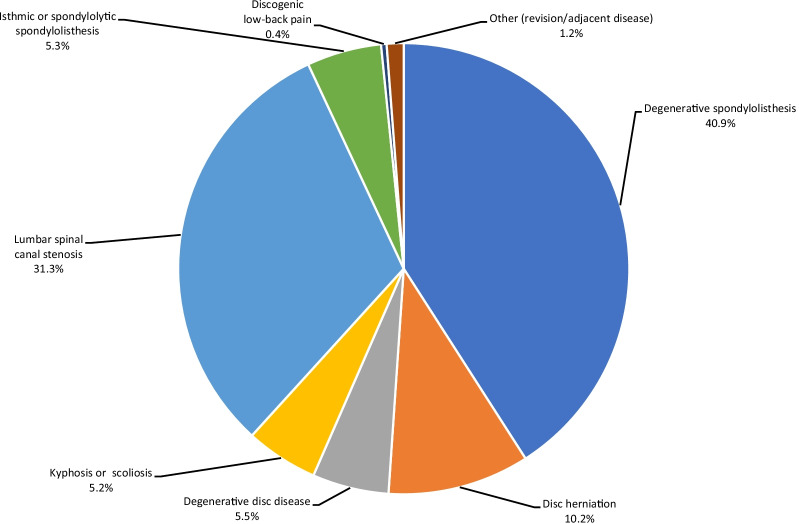
Major diagnoses and their proportions for 22 included articles

There were 22 studies provided pre-operative diagnosis of involved patients, which included spondylolisthesis (degenerative, ischemic, or spondylolytic), disc herniation, DDD, kyphosis, scoliosis, spinal canal stenosis, discogenic low back pain, and other reasons such as revision [20, 25, 31–35, 37–39, 42–44, 46, 48, 51–56, 81]. The corresponding patient distributions for these diagnoses are plotted in Fig. 2. The dominant condition associated with fusion surgery was degenerative spondylolisthesis (40.9%), followed by lumbar spinal canal stenosis (31.3%). More detailed demographics and diagnosis are summarised in Additional file 1.

### Anatomical and surgical factors

#### Fusion level and multi-level fusion

Details of fusion levels were provided in 21 of the 29 included articles, describing 7,355 cages [20, 25, 31, 32, 34–39, 41–43, 45, 47, 49, 51–53, 55, 56]. Qualities ranged from low (*n* = 1), moderate (*n* = 16), and high (*n* = 4). The distribution of fusion levels was as follows: L1/L2: 90 (1.2%); L2/L3, 426 (5.8%); L3/L4: 1,261 (17.1%); L4/L5: 3,852 (52.4%); and L5/S1: 1,681 (22.9%). Cage subsidence was identified in 620 cages, but the fusion level was only provided for 289 of them [20, 32, 45, 49, 52, 53, 55], with the following distribution: L1/L2, 2 (0.7%); L2/L3, 8 (2.8%); L3/L4, 35 (12.1%); L4/L5, 184 (63.7%); and L5/S1, 60 (20.8%). According to these seven studies, the subsidence rates for cages at distal levels (L4/L5 and L5/S1) ranged from 7.4 to 62.2%, and the total subsidence rate was 27.7%. Similarly, cage migration was noted in 116 cages, but the distribution of fusion levels was only provided for 103 of them: L2/L3, 2 (1.9%); L3/L4, 7 (6.8%); L4/L5, 56 (54.4%); and L5/S1, 38 (36.9%) [25, 38, 41, 42, 47, 56]. For fusion at distal levels, cage migration rates from these five studies ranged from 0.7 to 18.2%, and the total migration rate was 2.4%. Three studies reported fusion surgery at distal levels as a risk factor for cage subsidence or migration after LIF [20, 37, 47] and found higher adverse event rates for fusion at distal levels (*p* < 0.05 or odds ratio > 1).

Thirteen studies provided information regarding single- and multi-level fusion (fusion level $\ge$ 2) for all patients and patients with adverse events [20, 31, 33, 34, 40–42, 47–49, 53, 54, 56]. Among the 3181 patients in these studies, 2311 had single-level fusion (72.7%) and 870 had multi-level fusion (27.3%). The cage subsidence rates for single-level fusion and multi-level fusion were 15.5% and 26.2%, respectively, and the posterior cage migration rates were 4.9% and 5.3%, respectively. Although the cage subsidence rate of multi-level fusion was higher than that of single-level fusion, to the best of our knowledge, no study has shown the significance of multi-level fusion on cage subsidence. Three studies mentioned multi-level fusion as a significant risk factor (*p* < 0.05) for posterior cage migration after LIF [25, 38, 54].

#### BMD

Six studies evaluated the magnitude of BMD in patients with and without adverse events [34, 46, 48–50, 53], of which four were of moderate quality, whereas the remaining studies were of high quality [48, 53]. Two of these studies also accounted for osteoporosis (T-score < − 2.5) [34, 48]; Cho et al. [34] included 86 patients and reported that the cage subsidence rates after LIF were significantly higher for osteoporotic patients than for non-osteoporotic patients (65.4% and 17.6%, respectively; *p* < 0.001). Park et al. [48] found that the adverse event rates of cage migration cases and combined cage subsidence and migration cases were 9.7% and 18.1% for osteoporotic patients (72), respectively, whereas the rates were 2.6% and 4.6% for all patients (*n* = 784) in the study. Yao et al. [53] evaluated 126 cages used for LIF, the mean T-score for 43 cages with subsidence was − 1.8, whereas that for 83 cages without subsidence was − 1.1. Singhatanadgige et al. [49] included 135 cages used for TLIF, the mean T-score for 80 cages with subsidence was − 1.3, whereas that for 55 cages without subsidence was − 1.1. Tempel et al. [50] evaluated 80 patients with LIF, wherein 39 (48.8%) had decreased BMD (T-score < − 1), and for 23 patients with cage subsidence, 18 (78.3%) had decreased BMD. Okuyama et al. [46] evaluated 52 patients with LIF, with a mean BMD of 0.879 g/cm^2^. For 12 patients with non-union or undetermined union, the mean BMD was 0.674 g/cm^2^ and 0.710 g/cm^2^, respectively. The findings of these studies identified a trend of higher subsidence rates in patients with decreased BMD and osteoporosis. In addition, one study with moderate quality mentioned patients with cage subsidence had significantly low Hounsfield unit (HU) of their CT scans (113.4 ± 10.5, *p* = 0.0075) [45], and low HU of CT scans was associated with low BMD. Xi et al. [52] held the same point in their study because the mean HU of patients who experienced cage subsidence was 20.8% lower than the mean HU of patients without cage subsidence.

#### Pre-operative disc height

Six studies involving cage migration mentioned the disc height of the involved patients [25, 33, 38, 41, 42, 56], which were of moderate quality. Each study measured the disc height before and after fusion surgery, and some studies measured both anterior and posterior disc height. Patients with and without migration had similar average pre-operative disc height, whereas Yao et al. [53] found that shorter disc height was statistically significant for cage subsidence (*p* = 0.002).

#### Disc morphology

**Fig. 3:**
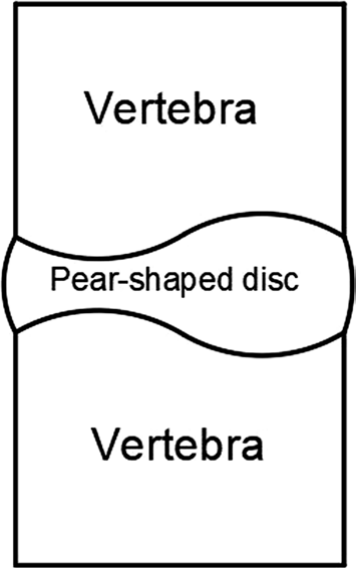
Sketch of pear-shaped disc (left side is anterior direction)

Five included studies evaluated pear-shaped/irregular disc as a risk factor for cage migration and cage subsidence after LIF [38, 41, 48, 55, 56] (Fig. 3), with qualities of moderate (*n* = 4) and high (*n* = 1). For 96 cages that underwent migration or subsidence in these studies, 26 (27.1%) were inserted into a pear-shaped/irregular disc, and 165 (6.6%) were inserted into pear-shaped/irregular disc for patients without cage subsidence or migration. Regarding the incidence of combined adverse events (cage migration + cage subsidence), the third study reported eight pear-shaped discs (22.2%) at all levels with combined adverse events, and the rate dropped to 6.2% for all levels without adverse events after LIF.

#### Lordosis

Five studies, all of moderate quality [32, 39, 44, 55, 56], reported pre-operative segmental lordosis in patients who received LIF. Marchi et al. [44] recorded the pre-operative segmental lordosis of 74 patients who underwent LIF, wherein the mean lordosis for patients who experienced obvious cage subsidence and for patients experiencing only slight subsidence or no subsidence was 49.1° and 47.6°, respectively. Four studies reported small differences (maximum difference 0.6°) between pre-operative segmental lordosis in patients with and without adverse events after LIF [32, 39, 55, 56].

#### Range of motion

For the pre-operative range of motion (ROM) of the lumbar segments, two studies, one of moderate and high quality each, reported a larger average ROM for patients with adverse events than patients without adverse events (migration: 10.7° vs. 5.6°; 9.6° vs. 7.7°; migration + subsidence: 8.1° vs. 7.7°) [38, 48]. However, two other studies, both of moderate quality, recorded slightly lower average ROM for patients with cage migration than those without cage migration (7.3° vs. 8.8° and 7.6° vs. 8.1°, respectively) [33, 56].

#### Screw fixation

Two moderate-quality studies have evaluated the influence of unilateral and bilateral screw fixation on cage migration [43, 81]. In these studies, 155 patients underwent unilateral pedicle screw fixation and 162 patients underwent bilateral pedicle screw fixation, with migration rates of 12.3% and 3.7%, respectively. Both these studies found that unilateral screw fixation was a significant risk factor for cage migration (*p* < 0.05).

### Cage specifications

#### Cage material

All articles except four [41, 42, 45, 50] mentioned the material details of the spinal cages involved in their studies (qualities: low/moderate/high, 2/18/5). Of the 13 studies that evaluated cage subsidence, there were 2236 cages and the proportions of cage material were as follows: titanium, 174 (7.8%); porous titanium, 46 (2.1%); and PEEK, 2016 (90.2%) [20, 31, 32, 34, 36, 37, 40, 44, 49, 51–53, 55]. There were 678 subsided cages with the following materials: titanium, 18 (2.7%); porous titanium, 20 (2.9%); and PEEK, 640 (94.4%). Of the 10 studies focused on posterior cage migration after LIF, there were 5,244 cages and the proportions of cage material were as follows: titanium, 910 (17.4%); porous titanium, 5 (0.1%); PEEK, 4,312 (82.2%); and carbon, 17 (0.3%) [25, 33, 35, 38, 43, 47, 48, 54, 56, 81]. There were 130 cages that underwent migration, and the cage material proportions changed to titanium: 4 (3.1%) and PEEK: 126 (96.9%). There was no comparison between the different cage materials in the two studies that reported non-union after LIF.

#### Cage size

Four studies, three of moderate quality [33, 42, 49] and one of low quality [54], investigated the influence of cage size on posterior cage migration. Aoki et al. [33] evaluated the effect of cage size by calculating the difference between the cage height and pre-operative disc height. Migrated cages were 2.3 mm thinner (anterior) or 0.8 mm taller (posterior) compared with the disc, whereas for non-migrated cages, the cages were 2.5 mm taller (anterior) or 5 mm taller (posterior) than the disc. These distributions illustrated that undersized cages were a risk factor for cage migration (all *p* < 0.01). The details (sample size, grouping method, and complication rates) of the other three studies were compared (Table 5).

**Table 5:** Details of studies that evaluated effect of cage size on adverse events

Article	Li et al. [42]			Singhatanadgige et al. [49]			Zhao et al. [54]	
Number of total patients (adverse event rate)	286 (5.6%)			135 (59.3%)			512 (1.2%)	
Adverse event type	Migration			Subsidence			Migration	
Grouping method	Three levels of cage height			Three levels of cage height			Small cage (smaller than 28 mm × 14 mm × 9 mm) and large cage (larger than 31 mm × 18 mm × 11 mm)	
Group type	11 mm	12 mm	13 mm	8 mm	10 mm	12 mm	Small cage	Large cage
Number of patients with specific cage size (adverse event rate)	16 (50.0%)	60 (10.0%)	210 (1.9%)	24 (45.8%)	63 (54.0%)	48 (72.9%)	78 (5.1%)	434 (0.5%)

Three studies, two of moderate [40, 44] and one of high quality [51], evaluated the effect of cage width on cage subsidence. Upon pooling the data across these three studies, there were 313 and 242 cages with 18 mm and 22 mm width, respectively, and the incidence of cage subsidence was as follows: 87 cases (27.8%) of 18-mm width cages, and 28 cases (11.6%) of 22-mm width cages.

#### Shape

Five studies mentioned the influence of cage shape on posterior cage migration after LIF and classified cages as either straight (either bullet-shaped or rectangular-shaped) or curved (either wedge-shaped or banana-shaped) [33, 43, 47, 54, 56]. Qualities ranged from low (*n* = 2) and moderate (*n* = 3). Of the 981 patients undergoing fusion surgery, 383 had straight cages and 646 had curved cages. There were 27 (7.0%) migrated cases for the straight cage and 20 (3.1%) migrated cases for the curved cage. Two studies further evaluated the effect of cage shape by dividing cages into open and closed box cages. One moderate-quality study involving 46 closed and 34 open box cages described an incidence of subsidence in 20 (43.5%) and 4 patients (11.8%), respectively [31]. Another study of moderate quality that incorporated open or closed box designs did not find that any of the five closed box cages migrated, whereas nine out of 1,433 open box cages did experience migration (0.6%) [38].

#### Position

Five articles, three of moderate [31, 32, 49] and two of high quality [48, 52], have reported cage positions in their studies. Of these studies, 388, 874, and 67 cages were located at the anterior, posterior, and medial position of the disc space, respectively. The incidence of cage subsidence or migration for these cage positions was anterior (26.0%), posterior (19.1%), and medial (50.7%). Four of these studies showed that cages located at the medial or posterior position had higher adverse event rates (*p* < 0.05) [32, 48, 49, 52].One study of moderate quality found that more subsided cages were located at the posterior position of the disc space (10 of 14) [20]. Two other moderate-quality studies evaluated the influence of cage position by calculating a depth ratio, defined by the distance between the cage centre and disc centre divided by the lateral width of the endplate. A lower depth ratio indicated that the cage was located more posteriorly. Univariate analysis of these studies indicated that migrated cages had a significantly lower depth ratio (*p* < 0.001) [25, 56].

### Summary of risk factors

**Fig. 4:**
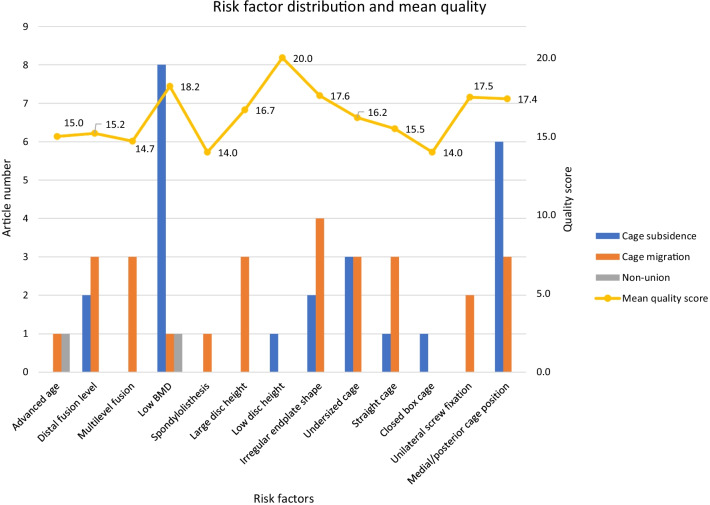
Distribution of risk factors for adverse events after LIF and corresponding mean quality scores

The main risk factors of adverse events after LIF that are associated with the endplate were advanced age, distal fusion level, multi-level fusion, low BMD, spondylolisthesis, disc height, irregular endplate shape, undersized cages, straight cages, closed box cages, unilateral screw fixation, and medial/posterior cage position on the endplate. The number of articles focused on each risk factor for different adverse events after LIF and the corresponding mean quality scores are summarised in Fig. 4.

Risk factors such as low BMD, medial/posterior cage position, irregular endplate shape, undersized cage, and distal fusion level were the most frequently reported factors. The mean quality score of a risk factor is the average quality score of all articles involving this risk factor. The mean quality scores of all included risk factors are $\ge$ 14 (moderate quality), which means the findings associated with each risk factor have at least modest reliability.

**Fig. 5:**
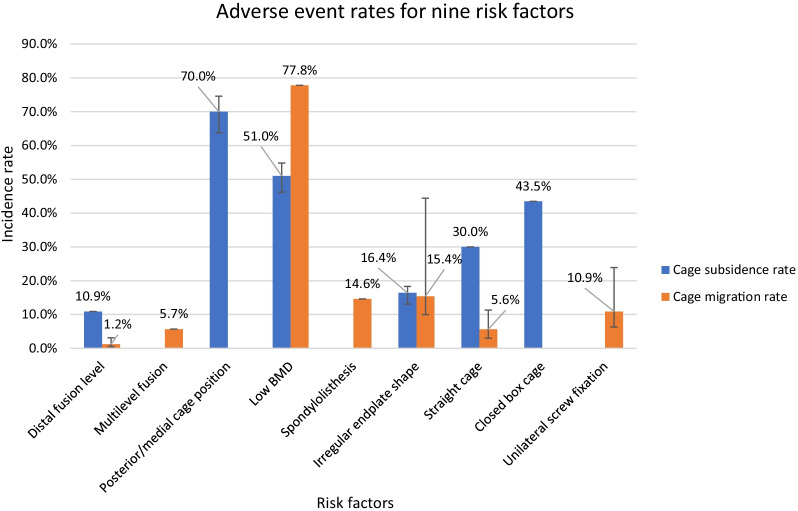
Different risk factors’ adverse event rates when the sample size changed to the number of cages that were associated with these risk factors. If the incidence rate was calculated based on more than one study, the error bar shows the range of adverse event rate

The incidence rates for cage subsidence and cage migration were only available for nine risk factors (Fig. 5).

## Discussion

This systematic review aimed to identify how geometric and material property factors contribute to adverse outcomes following LIF. A total of 29 studies described four primary adverse events: cage subsidence, cage migration, combined cage subsidence and migration, and non-union. Interbody device subsidence was associated with an increased fusion failure rate [11], and screw loosening, as one of the causes of non-union, can lead to a higher fusion failure rate [34], while other risk factors may contribute to fusion failure, including demographics and cage specifications.

**Fig. 6:**
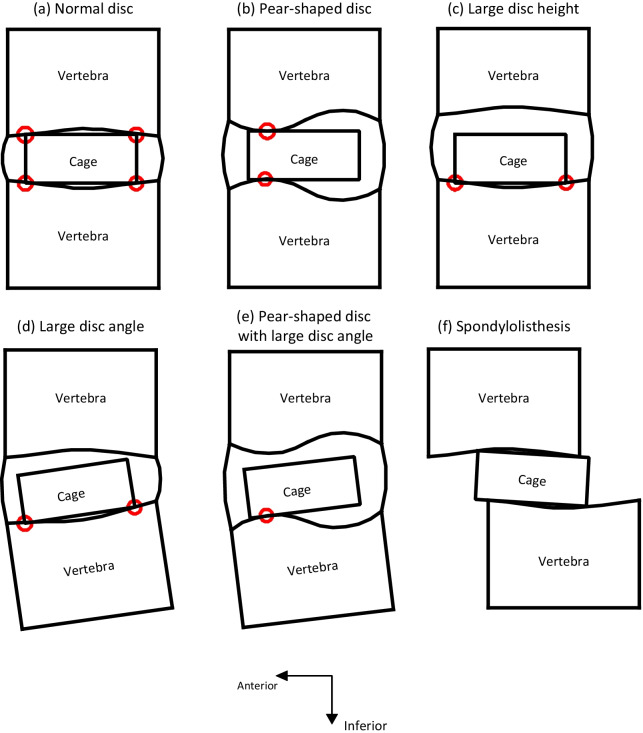
Different contact scenarios of fusion level when cage inserted. **a** The interbody cage inserted in a normal intervertebral disc space; **b** cage inserted in a pear-shaped disc space; **c** cage inserted in a disc space having large height; **d** cage inserted in a disc space having large angle; **e** cage inserted in a pear-shaped disc space having large angle; **f** cage inserted in a disc space having spondylolisthesis

Among all the geometric features of the lumbar endplate, a pear-shaped disc was the most common risk factor for adverse events after LIF. The pear-shaped disc is described as having both endplates with convex surfaces in its anterior halves and concave surfaces in its posterior halves (Fig. 6). For a normal intervertebral disc, both endplates have a slightly concave surface, indicating that there would be at least four contact points between the interbody cage and endplates after inserting the common cage into the intervertebral space (Fig. 6a). However, the contact regions can be decreased to two if the same cage is inserted into the space of the pear-shaped disc (Fig. 6b), and the contact forces would ultimately act on one side of the interbody cage, which might cause movement of the cage. This phenomenon was a significant risk factor for negative events after LIF for pear-shaped discs [38, 41, 47, 48, 56].

Other factors can also reduce contact points between the cage and endplates, possibly leading to cage motion. For instance, a large disc height can result in the lack of superior contact forces on the interbody cage, which generated instability after LIF (Fig. 6c). Therefore, large disc height [25, 33] and undersized cage [33, 42, 54] were shown to be contributors for adverse events after LIF. However, this cannot explain why shorter disc heights were observed with cage subsidence in another study [53]. This may relate to intraoperative technique as larger discs might be relatively undersized. A large disc angle (lordotic angle) may also cause instability after LIF due to decreased contact points, especially for a pear-shaped disc with a large disc angle as only one contact point may be preserved in this circumstance (Fig. 6d, e). In addition, a surgeon may have difficulty selecting an appropriate interbody cage for PLIF in this scenario because of the low posterior disc height [38]. Since the disc angle increases towards a lower level [15], LIF at distal levels may cause negative outcomes [20, 37, 38, 47, 83]. A similar unstable contact between cage and endplates was caused by spondylolisthesis, where one of the lower vertebrae generally slips anterior to the caudal vertebrae [84]. The contact interface between the cage and endplates is decreased due to slippage (Fig. 6f), leading to a higher rate of adverse events after LIF [42]. Because of the pre-existing slip, there are also generally higher abnormal shear forces presented at that level. Due to limited access to the original data associated with included studies, further analysis of the relationships between various patient pathologies and fusion failure was not feasible.

In addition to instability at the fusion site caused by insufficient contact between the cage and endplates, the relative movements of the endplates (vertebrae) can affect LIF outcomes. The interbody cage was more likely to move with a rotatory motion within the intervertebral space [81]. Accordingly, the lack of posterior fixation and unilateral pedicle screw fixation may not effectively restrict the rotatory motion between endplates, leading to higher rates of adverse events after LIF [81, 85]. However, unilateral pedicle screw fixation can reduce intraoperative blood loss and shorten the operation time [86, 87]. Therefore, surgeons may choose a screw fixation method in LIF based on the constraints imposed by the specific case.

BMD, strength, and thickness are critical material properties of the lumbar endplate. Endplate regions with low BMD/strength/thickness are vulnerable areas particularly susceptible to fractures. As osteoporosis is associated with decreased BMD, osteoporosis is a vital risk factor related to adverse events after LIF [34, 47, 48]. Okuyama et al. [46] provided a specific BMD value range that could increase the risk of non-union after PLIF, which is useful for interbody cage design and selection for LIF. For LIF involving posterior fixation, osteoporosis can lead to screw loosening [34]. In addition, similar to the principle mentioned previously, screw loosening would affect the ability of posterior instruments to restrict motion between the vertebrae, indicating associations between screw loosening and non-union [81]. Combined with the associations between cage retropulsion and screw loosening [41], the relationship between osteoporosis and adverse events after LIF is clear. As osteoporosis is more common in older people [88], advanced age is considered as a contributing factor for adverse events such as cage subsidence, cage migration, and non-union [35, 39, 49, 55].

**Fig. 7:**
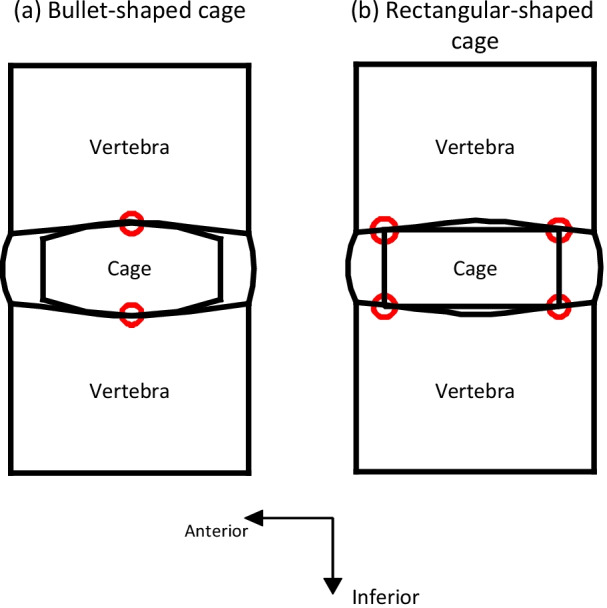
Two contact scenarios of fusion level when straight cages inserted. **a** A bullet-shaped cage inserted in an intervertebral disc space; **b** a rectangular-shaped cage inserted in a disc space

Compared with the peripheral region of the endplate, the central area of the lumbar endplate is thinner and weaker [19, 23, 24]. Therefore, the influence of the contact area location between the interbody cage and the endplate should be considered. For example, a cage placed at a medio-medial position has been shown to lead to a high migration rate after LIF [31]. LIF using a unilateral single cage has also been shown to lead to a higher cage retropulsion rate than that of surgery with bilateral double cages because unilateral single cages tend to be located more centrally [48]. Similarly, because the peak of the concave surface of the endplate was the contact point between the bullet-shaped cage and adjacent endplate (Fig. 7a), this region is prone to endplate fractures because of the thinner and weaker central area of the endplate.

Use of straight cage (bullet-shaped cage and rectangular-shaped cage) was found to be a risk factor for adverse event after LIF [33, 47, 76, 89]. Except the material properties of the endplate region in contact with bullet-shaped cage, the contact points cannot generate offset forces to stabilise the fusion level (Fig. 7a) [33]. Although the rectangular-shaped cage could have the ability to resist the mechanical forces to push the cage out, the small contact area between this type of cage and endplates could create high stress concentrations on the endplates (Fig. 7b), which was adverse for bony fusion [90].

Although the screw fixation method and cage specifications did not belong to geometric and material properties of the endplate, these factors were included because they could influence the interbody cage motion by changing cage-endplate contact area, angle, and site, which were associated with endplate geometric and material properties.

To prevent the postoperative adverse events of LIF, the following recommendations were drawn for cage design or selection based on the results of this review: (1) employ a cage with a relatively large contact area with endplates to eliminate stress concentrations on the endplates; (2) use a cage with a height or intervertebral angle that maximises contact area between the cage and endplates; (3) preferably, this contact area is concentrated in the periphery of the disc space to take advantage of the best quality bone; (4) select bilateral pedicle screw fixation if the surgeon aims to minimise inadvertent micro-motions inside the disc space; (5) carefully select interbody cage and counsel for patients who are elderly, have osteoporosis, and have certain spinal disorders.

Several systematic reviews on LIF have focused on three aspects: comparison between different types of LIF, influence of surgical factors on LIF, and effectiveness of different devices used in LIF [91–93]. However, only few review papers have directly investigated the risk factors for adverse events after LIF, and these studies were limited to only one or two types of postoperative adverse events. Therefore, the number of included articles was an inevitable limitation of the previous review on risk factors [89]. Based on similar reasons behind different adverse events after LIF (especially cage migration and cage subsidence), some risk factors for different postoperative adverse events were grouped and analysed together in this systematic review. This provides a more comprehensive interpretation of the influence of different risk factors on adverse events after LIF.

Our systematic review has some limitations. First, the data reported in all included articles was heterogeneous, or limited in sample size or data output, which meant that a meta-analysis was not practical. Consequently, the risk factors for LIF in this review were not statistically analysed. Second, the number of articles included was relatively small. Hence, there may be some risk factors that have not been covered in this systematic review, or some risk factors illustrated in this review were not fully evaluated. However, previous systematic reviews focusing on LIF results had similar numbers of included articles [89, 92, 94, 95]. Third, the validity of the customised checklist for quality assessment was not evaluated; however, the questions of the customised checklist were derived from the STROBE statement and Downs and Black checklists which were deemed suitable for judging methodological quality of observational, randomised, and non-randomised studies [29, 30]. Finally, computational works were excluded in this review because we specifically focused on available clinical data regarding post-operative LIF complications. There are, however, computational and 3D morphometric studies that have analysed geometric and bone density-related factors of the lumbar endplate [96–98]. These studies showed variations of endplate shape across lumbar levels and influence of cage positions on fusion outcomes and may be useful in informing the design of future interbody devices.

## Conclusions

This systematic review provided a summary of published studies that focused on risk factors for cage subsidence, cage migration, combined cage subsidence and migration, and non-union after LIF. Of particular interest to us was to investigate fusion failure risk factors associated with geometric and material properties of the endplate. This is an area which is relatively less explored in the literature, primary risk factors included advanced age, osteoporosis, spondylolisthesis, undersized or straight cage, medial or posterior cage position, irregular endplate shape, distal fusion level, and multi-level fusion. These factors were associated with reduced cage and endplate contact interface, thin and weak bony endplates, and spinal diseases that weaken the endplate and vertebrae or decrease the contact surface between the cage and endplate. Further studies are required to analyse the significance of all the main risk factors for adverse events after LIF based on accurate data from the same group of patients. The results of this review study may help guide device selection and surgical decision making in LIF.

## Supplementary Information

**Additional file 1**. Reasons for setting up exclusion criteria, quality assessment checklist, quality score distribution, and detailed demographics and diagnosis for included patients.

**Additional file 2**. The PRISMA 2020 checklist for this systematic review.

**Additional file 3**. Results of quality assessment on each included study.

**Additional file 4**. Available data of patients involved in included studies.

## Data Availability

The data generated and/or analysed during the current study are available from the corresponding author on reasonable request.

## Abbreviations

ALIF: Anterior lumbar interbody fusion
BMD: Bone mineral density
BMI: Body mass index
DDD: Degenerative disc disease
HU: Hounsfield unit
LIF: Lumbar interbody fusion
LLIF: Lateral lumbar interbody fusion
MIFTLIF: Minimally invasive transforaminal lumbar interbody fusion
PEEK: Polyetheretherketone
PRISMA: Preferred Reporting Items for Systematic Reviews and Meta-Analyses
PLIF: Posterior lumbar interbody fusion
ROM: Range of motion
STROBE: Strengthening the Reporting of Observational Studies in Epidemiology
TLIF: Transforaminal lumbar interbody fusion
XLIF: Extreme lateral lumbar interbody fusion
