# Supplementary material for: Influence of the geometric and material properties of lumbar endplate on lumbar interbody fusion failure: a systematic review
Source: J Orthop Surg Res. 2022 Apr 10;17:224. doi: 10.1186/s13018-022-03091-8 (PMC8996478; doi:10.1186/s13018-022-03091-8)
Supplement: Supplementary file 1 — Additional file 1. Reasons for setting up exclusion criteria, quality assessment checklist, quality score distribution, and detailed demographics and diagnosis for included patients. [file 13018_2022_3091_MOESM1_ESM.docx]

**Supplementary Material**

**Reasons for setting up exclusion criteria**

Table S1 shows the detailed reason for setting up exclusion criteria.

*Table S1: Reasons for setting up exclusion criteria*

| **Exclusion criteria** | **Reason** |
| --- | --- |
| Animal studies | Animal spine has different geometry and material properties compared to human spine. |
| Finite element (computational) studies | They are not correlated with clinical data necessarily. It is difficult to stimulate fusion failure in finite element studies. |
| Rare case studies | Rare case studies typically do not have sufficient data to be of statistical significance in a large systematic review. |
| Previous systematic review/meta-analysis | This review focused more on clinical data, previous systematic review or meta-analysis is a secondary literature. |

*Table S2: Quality assessment checklist for the systematic review*

| **Quality assessment checklist** | |
| --- | --- |
| 1 | An informative abstract was clearly described. |
| 2 | The explanation of scientific background and rationale was reported. |
| 3 | The specific objective/aim with hypothesis was clearly stated. |
| 4 | The characteristics of the cases included in the study along with inclusion and exclusion criteria was clearly described. |
| 5 | The measured parameters and measurement methods were clearly introduced. |
| 6 | The main results were clearly expressed. |
| 7 | The actual probability values of clinical data were reported for main outcomes. |
| 8 | The potential error of the data was clearly stated. |
| 9 | The appropriate overall interpretation of results was given. |
| 10 | Considering potential bias, the limitations of the study were discussed. |
| 11 | The probable application of the study results was discussed. |
| 12 | The source of funding and role of funders or other support (e.g. data supply) was clearly described. |

*Figure S1: Quality score distribution for all included articles in the systematic review.*

**Detailed demographics and diagnosis**

**Age**

The mean age of the 7,070 patients in all included studies was 60.0 years (range, 18.0-86.0 years). There were 17 of the included studies that provided the age distribution of patients experiencing adverse events after LIF [25, 32, 34, 38, 41, 42, 44, 45, 47-50, 52, 53, 55, 56, 81]. Among these studies, three were of high quality [48, 52, 53], 13 of moderate quality [25, 32, 34, 38, 41, 42, 44, 45, 49, 50, 55, 56, 81], and one of low quality [47]. Patients with cage subsidence after LIF had mean age of 61.6 years (range: 35.0 - 80.5 years) based on nine studies [32, 34, 44, 45, 49, 50, 52, 53, 55]. The mean age of patients with posterior cage migration was 60.0 years (range: 27.0 - 79.0 years) according to the other eight studies [25, 38, 41, 42, 47, 48, 56, 81]. For patients with combined cage migration and subsidence, Park et al. [48] specified a mean age of 65.5 years. No age details were provided for patients with non-union after LIF. Two studies mentioned old age as a risk factor for cage subsidence after LIF [49, 55], whereas other two mentioned old age as a contributor for cage migration and non-union after LIF [35, 39], and these four studies reported significantly higher adverse event rates for older patients (P<0.05).

**Body mass index**

Seven studies included the body mass index (BMI) of patients receiving LIF along with patients who experienced adverse events [32, 41, 44, 49, 53, 55, 56]. One of these studies was of high quality [53] and the other six were of moderate quality [32, 41, 44, 49, 55, 56]. The mean BMI for the 1,459 patients involved in these seven studies was 24.9 kg/m^2^. The mean BMI of patients with posterior cage migration ranged from 22.8 kg/m^2^ to 24.2kg/m^2^ [41, 56], and the mean BMI of patients with cage subsidence ranged from 24.4 kg/m^2^ to 27.5 kg/m^2^ [32, 44, 49, 53, 55].

**Sex**

A total of 16 studies provided the sex distribution of patients who experienced adverse events after LIF [25, 32, 34, 38, 41, 42, 44, 47-50, 52, 53, 55, 56, 81]. The quality of these studies ranged from high (n=3) [48, 52, 53], moderate (n=12) [25, 32, 34, 38, 41, 42, 44, 49, 50, 55, 56, 81], and low (n=1) [47]. Among these studies, nine investigated cage subsidence [32, 34, 44, 48-50, 52, 53, 55], and eight investigated posterior cage migration [25, 38, 41, 42, 47, 48, 56, 81], noting that Park et al. [48] considered the combined occurrence of these adverse events. In the studies involving cage subsidence, sex distribution of the 1,612 patients who received LIF was 35.8% male and 64.2% female, whereas among the 411 patients identified with cage subsidence, 28.2% were male and 71.8% were female [32, 34, 44, 48-50, 52, 53, 55]. For studies about posterior cage migration, 4,068 patients received LIF, with sex distribution being 43.1% male and 56.9% female, and of the 133 patients who experienced posterior migration, 47.4% were male and 52.6% were female [25, 38, 41, 42, 47, 48, 56, 81].

**Osteoporosis**

Three studies accounted for patients with osteoporosis [34, 41, 48]. Of these three studies, two articles, one of moderate and one of high quality, showed that osteoporosis may increase the rate of cage subsidence and cage migration after LIF [34, 48], whereas the third moderate-quality study showed no relationship between osteoporosis and the rate of adverse events [41]. Cho et al. [34] included 86 patients and reported that the cage subsidence rates after LIF were significantly higher for osteoporotic patients than for non-osteoporotic patients (65.4% and 17.6%, respectively; p<0.001). Park et al. [48] found that the adverse event rates of cage migration cases and combined cage subsidence and migration cases were 9.7% and 18.1% for osteoporotic patients (72), respectively, whereas the rates were 2.6% and 4.6% for all patients (n = 784) in the study. Lee et al. [41] showed that of 75 patients with osteoporosis (10% of the study participants), none experienced posterior cage migration.

**Spinal diseases**

Among the included studies, 22 provided pre-operative diagnosis of involved patients, which included spondylolisthesis (degenerative, ischemic or spondylolytic), disc herniation, DDD, kyphosis, scoliosis, spinal canal stenosis, discogenic low back pain and other reasons such as revision [20, 25, 31-35, 37-39, 42-44, 46, 48, 51-56, 81]. The corresponding patient distributions for these diagnoses are plotted in Figure 2. The dominant condition associated with fusion surgery was degenerative spondylolisthesis (40.9%), followed by lumbar spinal canal stenosis (31.3%). Only Li et al. [42] found spondylolisthesis as a contributing factor to cage migration (p=0.009).
